# Targeting Mnks for Cancer Therapy

**DOI:** 10.18632/oncotarget.453

**Published:** 2012-03-03

**Authors:** Jinqiang Hou, Frankie Lam, Christopher Proud, Shudong Wang

**Affiliations:** ^1^ School of Pharmacy and Medical Sciences, University of South Australia, Adelaide, Australia; ^2^ Centre for Biological Sciences, University of Southampton, Southampton, UK

**Keywords:** eIF4E, Mnk, Ras, Raf, MAPK, Akt, PI3K, mTOR, Targeted Cancer Therapy, Structure based drug design, Mnk Inhibitors

## Abstract

Deregulation of protein synthesis is a common event in human cancer and a key player in translational control is eIF4E. Elevated expression levels of eIF4E promote cancer development and progression. Recent findings suggest that eIF4E activity is a key determinant of the PI3K/Akt/mTOR and Ras/Raf/MEK/ERK mediated tumorigenic activity and targeting eIF4E should have a major impact on these pathways in human cancer. The function of eIF4E is modulated through phosphorylation of a conserved serine (Ser209) by Mnk1 and Mnk2 downstream of ERK. While the phosphorylation event is necessary for oncogenic transformation, it seems to be dispensable for normal development. Hence, pharmacologic Mnk inhibitors may provide non-toxic and effective anti-cancer strategy. Strong circumstantial evidence indicates that Mnk inhibition presents attractive therapeutic potential, but the lack of selective Mnk inhibitors has so far confounded pharmacological target validation and clinical development.

## INTRODUCTION

Mechanism-based targeted cancer therapy represents the remarkable progress of the decades' research into mechanisms of cancer pathogenesis. Most cancer drugs developed to date have been directed toward specific molecular targets that are involved in one way or another in enabling particular capabilities of tumour growth and progression. Such specificity of action presents inhibitory activity against a target resulting in a clinical response with less of target toxicity. However, the clinical response is often followed by relapses. One interpretation is that a targeted therapeutic agent inhibiting a single target or pathway in a tumour may not be able completely to shut off tumorigenic capabilities due to a partially redundant network, allowing some cancer cells to survive or adapt to the selective pressure imposed by the therapy and eventually re-establish oncogenic functionality [1]. On the other hand, some multi-targeted inhibitors have contributed to the effectiveness for cancer treatment [2]. For example, Sorafenib has demonstrated an excellent clinical outcome and is approved for the treatment of patients with renal cell carcinoma and hepatocellular carcinoma. This has been attributed to the broad specificity of Sorafenib, which inhibits other targets besides Raf, including VEGFR, Flt-3, PDGFR and others. Co-targeting the key components of several signalling pathways simultaneously has been proposed as a more effective drug development strategy [3].

Eukaryotic initiation factor 4E (eIF4E) is a general translation factor, but it has the potential to enhance preferentially the translation of messenger RNAs (mRNAs) that lead to production of a malignancy-associated proteins. This selectivity may relate to an increased requirement for eIF4E and its binding partners for the translation of mRNAs containing extensive secondary structure in their 5'-untranslated regions (5'-UTRs) [4-6]. These mRNAs include those encoding certain proteins that control cell cycle progression and tumourigenesis such as c-Myc and cyclin D1; growth factors (the basic fibroblast growth factor 2, FGF2 and vascular endothelial growth factor, VEGF), powerful promoters of cell growth and angiogenesis, as well as the anti-apoptotic protein Mcl-1 [6-12]. Under normal cellular conditions the translation of these malignancy-associated mRNAs is suppressed as the availability of active eIF4E is limited; however, their levels can increase when eIF4E is over-expressed or hyperactivated.

Elevated levels of eIF4E have been found in many types of tumours and cancer cell lines including cancers of the colon, breast, bladder, lung, prostate, gastrointestinal tract, head and neck, Hodgkin's lymphomas and neuroblastomas, but not in typical benign lesions [8, 10, 13-20]. A role for eIF4E as a prognostic marker has also been suggested for certain cancers and the involvement of eIF4E in metastasis has been considered [8, 10, 12, 21, 22]. Further evidence supporting a role for eIF4E in malignancy has been provided by studies where expression of antisense RNA to eIF4E in HeLa cells suppressed proliferation and altered cellular morphology [23]. Antisense RNA-mediated reduction of eIF4E in breast, head and neck cancer cells was also shown to suppress tumour formation, growth and metastasis [24-29]. Elevated eIF4E accelerated lymphomagenesis and promoted drug resistance in a transgenic mouse model [30]. The studies have provided proof of concept that the deregulation of eIF4E-mediated translation initiation is an important step in oncogenic transformation and may contribute to tumour maintenance.

**Figure 1:**
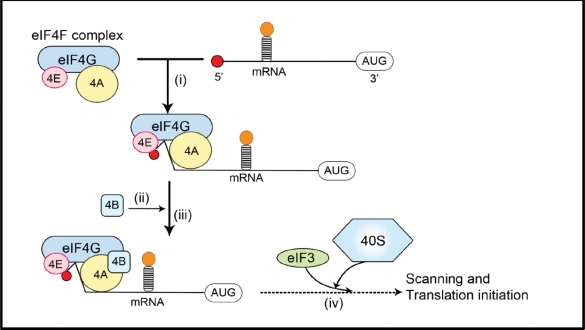
mRNA recruitment during translation initiation (i) The eIF4F complex including eIF4E, eIF4A and eIF4G is recruited to the mRNA via an interaction between eIF4E and the 5'-cap of the mRNA, which includes a 7-methylguanosine moiety; (ii) eIF4B binds to eIF4G and enhances the helicase activity of eIF4A; (iii) secondary structure (stem loops) in the 5'-untranslated region of the mRNA, which can inhibit translation initiation, are ‘unwound’ by eIF4A/eIF4B; (iv) the 40S ribosomal subunit is recruited to mRNA via an interaction between eIF4G and eIF3. Together with other translation factors, the 40S subunit then locates the start codon (‘AUG’) via ‘scanning’. The recruitment of the 40S subunit likely occurs earlier in the process than is depicted here.

Translation is tightly regulated. Initiation of cap-dependent translation is thought to depend on the assembly of eIF4F, an initiation factor complex including eIF4E, the scaffold protein eIF4G, and the RNA helicase eIF4A [31-33]. Because eIF4E is the only one of these proteins that binds directly to the mRNA cap structure, it is the key factor for the assembly of eIF4F at the 5' cap (Figure 1) [32, 34]. The scaffold protein, eIF4G, also recruits the 40S ribosomal subunit to the mRNA via its interaction with eIF3 and binds eIF4B, a protein that aids the RNA-helicase function of eIF4A, thus facilitating the translation of mRNAs that contain structured 5'-UTRs (Figure 1). The availability of eIF4E as part of the eIF4F complex is a limiting factor in controlling the rate of translation, and therefore eIF4E is an important regulator of mRNA translation. As described below, the availability of eIF4E is controlled by eIF4E-binding proteins (4E-BPs) which can interact with eIF4E and prevent it binding eIF4G. 4E-BPs undergo phosphorylation resulting in their release from eIF4E, allowing it to form eIF4F complexes.

**Figure 2:**
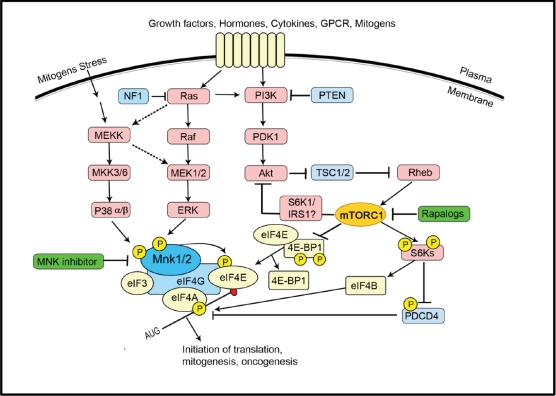
The regulation of eIF4E forms a node of convergence of two intracellular signalling pathways The Ras/Raf/ERK pathway culminates in the activation of the Mnks (especially Mnk1a). Mnk1a can also be activated by p38 MAPK downstream of cytokines or stressful stimuli. Mnk binds to eIF4G and phosphorylates eIF4E within the eIF4F complex. The functional consequences of phosphorylation of eIF4E are unclear but are believed to favour the translation of certain mRNAs. Stimulation of PI3K, e.g., by growth factors, initiates a cascade of events: PDK1 activates AKT which phosphorylates TSC2, thereby inactivating the TSC1/TSC2 complex. Rheb, the small G protein, is no longer inhibited by the GAP (GTPase-activating protein) activity of TSC2 and the resulting Rheb-GTP activates mTORC1 via unknown mechanisms. mTORC1 promotes the activation of the translational activator S6K and the hierarchical phosphorylation of 4E-BP. Hyperphosphorylated 4E-BP is released from eIF4E, thereby allowing eIF4E to bind to eIF4G. Activated S6K phosphorylates eIF4B and PDCD4, effects which promote the helicase activity of eIF4A. The dashed lines indicate possible links.

Regulation of eIF4E activity forms a node of convergence of the PI3K/Akt/mTOR and Ras/Raf/MAPK signalling pathways. A schematic overview of the signalling network is presented in Figure 2 [4, 32, 35]. The PI3K (phosphoinositide 3-kinase)/PTEN (phosphatase and tensin homologue deleted on chromosome ten)/Akt/mTOR (mammalian target of rapamycin) pathway is often involved in tumorigenesis and in sensitivity and resistance to cancer therapy. Deregulated signalling through the PI3K/PTEN/Akt/mTOR pathway is often the result of genetic alterations in critical components of this pathway and/or mutations at upstream growth factor receptors or signalling components. Activated by extracellular growth factors, mitogens, cytokines, receptors, etc., PI3K initiates a cascade of events. PDK1 activates Akt, which in turn phosphorylates and inactivates the tumour suppressor complex comprising TSC1 and 2 (tuberous sclerosis complex 1/2), resulting in the activation of mTORC1 (target of rapamycin complex 1) by Rheb-GTP. Activation of PDK1 and Akt by PI3Ks is negatively regulated by PTEN [2, 36]. PTEN is a critical tumour suppressor gene and is often mutated or silenced in human cancers [37-39]. Its loss results in activation of Akt and increases downstream mTORC1 signalling. The involvement of mTOR complex1 (mTORC1) in neoplastic transformation appears to depend on its regulatory role toward the eIF4F complex; overexpression of eIF4E can confer resistance to rapamycin [30]. mTORC1 regulates the eIF4F complex assembly that is critical for the translation of mRNAs associated with cell growth, prevention of apoptosis and transformation. mTORC1 achieves this by phosphorylation and inactivation of 4E-BPs and the subsequent dissociation of 4E-BPs from eIF4E (Figure 2). This then enables eIF4E to interact with the scaffold protein eIF4G permitting assembly of the eIF4F complex for the translation of structured mRNAs [34, 40, 41]. mTORC1 also promotes activation of the translational activator, S6K, which phosphorylates the ribosomal protein S6 and other substrates, including eIF4B [42]. mTORC1 signalling is inhibited by rapamycin and its analogues (rapalogs), although these compounds act allosterically, rather than directly inhibiting mTOR kinase activity. Rapamycin and its analogues have been shown to be cytostatic, not cytotoxic, to leukemic and other cancer cells.

Given the importance of the PI3K/Akt/mTOR pathway in regulating mRNA translation of genes that encode for pro-oncogenic proteins and activated mTORC1 signalling in a high proportion of cancers, these kinases have been actively pursued as oncology drug targets [43, 44]. A number of pharmacological inhibitors have been identified, some of which have reached advanced clinical stages [2, 45]. However, it has recently become clear that the mTOR pathway participates in a complicated feedback loop that can impair activation of Akt [30, 46, 47]. It has been shown that prolonged treatment of cancer cells or patients with mTOR inhibitors causes elevated PI3K activity that leads to phosphorylation of Akt and eIF4E, and promotes cancer cell survival [48, 49]. eIF4E, acting downstream of Akt and mTOR, recapitulates Akt's action in tumourigenesis and drug resistance, and Akt signalling via eIF4E is an important mechanism of oncogenesis and drug resistance *in vivo* [30]. For these reasons, dual targeting of both Akt and mTOR, or directly inhibiting eIF4E activity, have been proposed as treatments for cancer [2, 30, 50, 51].

In addition to the PI3K/Akt/mTOR pathway, eIF4E is also the target of the Ras/Raf/MAP signalling cascade which is activated by growth factors and for the stress-activated p38 MAP kinase pathway (Figure 2). Erk1/2 and p38 then phosphorylate MAP kinase-interacting kinase 1 (Mnk1) and Mnk2. The Erk pathway is also activated in many cancers, reflecting, for example, activating mutations in Ras (found in around 20% of tumour cells) or loss of function of the Ras GTPase-activator protein NF1.

Mnk1 and Mnk2 specifically phosphorylate serine 209 (Ser209) of eIF4E within the eIF4F complex, by virtue of the interaction between eIF4E and the Mnks, which serves to recruit Mnks to act on eIF4E [49, 52]. Mnk1 and Mnk2 knock-out or knock-in mice, in which Ser209 was replaced by alanine, showed no eIF4E phosphorylation and significantly attenuated tumour growth [53-55]. Significantly, while Mnk activity is necessary for eIF4E-mediated oncogenic transformation, it is dispensable for normal development [53]. Pharmacologically inhibiting Mnks may, therefore, present an attractive therapeutic strategy for cancer. Despite increased understanding of structure and function of the Mnks, little progress has been made with Mnk-targeted drug discovery. In this review we intend to update the progress made in validating the Mnks as a potential therapeutic target and to provide an insight into binding models of selected prototype inhibitors in complex with the Mnks. The rationales and inhibitor design principles will be discussed.

## STRUCTURE AND FUNCTIONS OF MNKS

**Figure 3:**
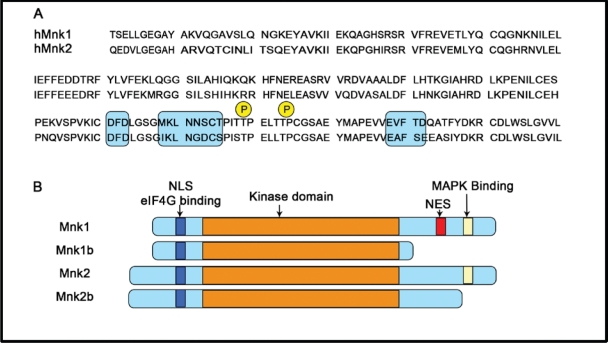
Schematic representation of the structure of splice variants of human Mnk1 and Mnk2 (A) Sequence alignment of kinase domains inserts the DFD motif (shaded box); Thr209/Thr214, the MAPK phosphorylation sites (P); and the kinase inserts (shaded box). (B) The layout indicates the arrangement of the known functional domains (as labelled). NLS: nuclear localization signal; NES: nuclear export signal; eIF4G, the scaffolding protein of the translation initiation complex eIF4F that binds Mnk1 and Mnk2.

Mnk1 and Mnk2 are threonine /serine protein kinases and were originally discovered as the result of screening for substrate s or binding partners for Erk [56, 57]. So far four human Mnk isoforms (Mnk1a, 2a, 1b and 2b) and two mouse Mnk isoforms (Mnk1and 2) have been reported [56-60]. Sequence alignment analysis reveals that all four isoforms have a nuclear localization signal (NLS) and an eIF4G-binding site in their N-terminal regions (Figure 3A) which, respectively, allow the kinases to enter the nucleus and to phosphorylate eIF4E efficiently. The central catalytic domains of the pairs of isoforms Mnk1a/b and Mnk2a/b are identical and closely homologous between Mnk1 and Mnk2 proteins [61]. The main structural differences lie within the C-terminal domain (Figure 3B). The C-terminal regions of Mnk1a and Mnk2a contain a MAPK-binding site, and thus can be phosphorylated and activated by Erk and p38 MAPK [49, 56]. Their short isoforms, Mnk1b and 2b, however, lack this domain and are poor substrates for Erk or p38 [58-61]. At least two threonine residues (Thr209 and Thr214 in human Mnks indicated in Fig. 3A) in this region are phosphorylated by MAPKs, and their replacement with alanine results in inactive kinases [56, 60, 62]. The threonine residues in Mnks correspond to the residues in MK2/3 (MAPK-activated protein kinases), which can also be phosphorylated by p38, suggesting a similar activation mechanism [63]. Furthermore, Mnk1a localises predominantly to the cytoplasm, whereas a significant proportion of the alternative Mnk variants is present within the nucleus. One possible explanation for this is that, although maintaining the NLS, these isoforms lack the C-terminal nuclear export sequence (NES) found in Mnk1a, impairing their exit from the nucleus to the cytoplasm [58, 62, 64, 65].While the activity of Mnk1a is tightly regulated by Erk and p38 MAP kinase, Mnk2a shows high basal activity, and Mnk1b and Mnk2b show, respectively, quite high and low activity, which appears to be unregulated, likely reflecting their lack of binding sites for Erk/p38 MAPK [65].

eIF4E is the only thoroughly-characterised physiological substrate for Mnks, although other substrates have been identified (reviewed in [65]), and expression of activated Mnks increases the cellular level of phosphorylated eIF4E in the cells [56, 66]. Both Mnk1 and Mnk2 specifically phosphorylate eIF4E at Ser209, and Ser209 is the only phosphorylation site in eIF4E [52, 55, 57, 67]. Mnk and eIF4E interact with eIF4G bringing them into physical proximity to facilitate eIF4E phosphorylation (Figure 2). The biological significance of eIF4E phosphorylation and its effect on translation is not completely understood. Biophysical studies indicate that phosphorylation of eIF4E actually decreases its affinity for the cap of mRNA, which play a role in facilitating scanning or permitting the transfer of eIF4E from mRNAs that are already undergoing translation to other mRNAs whose translation is subsequently promoted [49, 52].

In addition to its role in translation, eIF4E also appears to mediate the export of a set of mRNAs from the nucleus to the cytoplasm; these include mRNAs for a number of proteins involved in cell cycle progression or cell survival [68]. Phosphorylation of eIF4E by Mnks may also be important for its role in the export of some mRNAs, e.g., cyclin D [69] and hdm2 [70], providing a further mechanism by which phosphorylation of eIF4E may promote tumourigenesis.

*Drosophila* expressing a mutant eIF4E in which Ser251, the residue which corresponds to the Ser209 of mammalian eIF4E is mutated to alanine, show reduced viability [71]. By contrast, mice with deletions in both Mnk1 and Mnk2 develop normally without detectable eIF4E phosphorylation [30]. Recent studies confirmed that phosphorylation of eIF4E at the Ser209 by Mnk is essential for eIF4E's ability to promote tumourigenesis [72], while it is dispensable in normal tissue [21, 35, 54, 55, 72].

In an elegant study, a mouse model in which lymphomas generated from Eμ-Myc transgenic HSCs (hematopoietic stem cells) were transfected with wild-type *eIF4E* and *eIF4E*-mutants, was used to investigate their effects on oncogenicity [72]. Wild-type *eIF4E* greatly enhanced Myc-mediated lymphomagenesis compared to animals expressing eIF4E Trp56Ala, a mutant with defective cap-binding ability, implying a crucial oncogenic function for eIF4E. Similarly, mice reconstituted with cells carrying the Ser209Ala mutant were defective in tumour development to a similar extent to the Trp56Ala mice, suggesting that phosphorylation of Ser209 is important for eIF4E-mediated tumourigenesis. Conversely, activated Mnk1 promoted the onset of tumour development in a similar manner to eIF4E. *Mnk1*- and *eIF4E*-expressing lymphomas showed low levels of apoptosis compared to control tumours. This was attributed to the ability of eIF4E or Mnk1 to enhance the expression of the anti-apoptotic protein Mcl-1, and it was shown that Mnk1-mediated phosphorylation of eIF4E at Ser209 correlated with the level of Mcl-1 expression [72].

Further investigation of the link between Mnk1/2 and tumourigenesis driven by loss of PTEN demonstrated that Mnk1/2-double knock-out tPTEN^−/−^ mice (T-cell-specific PTEN conditional knockout mice) showed attenuated tumour growth compared to the parental tPTEN^−/−^ mice [54]. Phosphorylation of eIF4E was greatly enhanced in lymphomas from tPTEN^−/−^ mice compared with lymphoid tissues of wild-type mice, but was abolished in lymphomas of tPten^−/−^; Mnk1/2-double knock-out mice, confirming that Mnk1 and Mnk2 kinase activity are essential for eIF4E phosphorylation in transformed cells. This was consistent with the high levels of Mnk1 and eIF4E phosphorylation exhibited by human glioma U87MG cells bearing an inactivating PTEN mutation. Conversely, U87MG cells in which Mnk1 had been knocked down by shRNA showed substantially reduced levels of phosphorylated eIF4E and markedly decreased tumour formation [54]. A complementary study was carried out using knock-in mice, in which eIF4E Ser209 was mutated to alanine [55]. Mouse embryonic fibroblasts isolated from eIF4E Ser209A mice lacked eIF4E phosphorylation and displayed a marked resistance to transformation *in vivo*. The study failed to reveal any obvious phenotype in Mnk knock-in mice; however, cells derived from these mice are resistant to Ras-activated oncogenic transformation. All these studies provide the proof of concept that inhibition of Mnk activity may be an effective therapeutic strategy for selectively targeting cancer cells while sparing normal cells.

Several studies have shown that treatment of some types of cancer cells with rapamycin (or its analogs) actually increases the phosphorylation of eIF4E [51] which may promote tumourigenesis. This seems surprising, given that rapamycin should enhance the association of eIF4E with 4E-BPs and thus interfere with recruitment of eIF4E to the eIF4G/Mnk complex. However, rapamycin fails to inhibit 4E-BP1 phosphorylation in a number of cell types [73, 74]. Development of Mnk inhibitors may be of value in preventing these undesirable consequences of inhibiting mTORC1 using rapalogs.

## KNOWN MNK INHIBITORS

**Figure 4:**
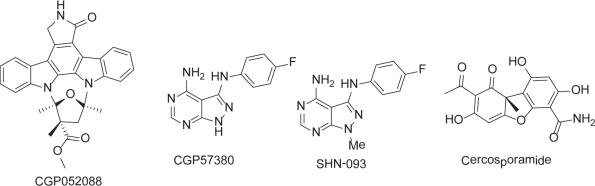
The chemical structures of MNK inhibitors and derivative

Despite increased understanding of Mnk structure and function, little progress has been made with the discovery of pharmacological Mnk inhibitors. So far three Mnk inhibitors have been reported: CGP052088 [75], CGP57380 [76-79], and Cercosporamide [80] (Figure 4). These compounds have mainly served as chemical biological tools for Mnk target validation.

CGP052088 is a derivative of staurosporine, a broad-spectrum kinase inhibitor. It inhibits Mnk1 with an IC_50_ value of 70 nM in biochemical assays and is cytotoxic with a GI_50_ value of 4.5 μM in a 24h-MTT proliferation assay [75]. CGP052088 blocked phosphorylation of eIF4E at Ser209 in human embryonic kidney 293 cells within 45 minutes. Interestingly, a closely related stereoisomer, CGP052428, failed to show a similar action. This was attributed to CGP052428 lacking Mnk1 inhibitory activity, although it has the same cellular cytotoxicity compared as CGP052088. Both compounds likely affect other enzymes in addition to the Mnks.

CGP57380 [81], 4-amino-3-(p-fluorophenylamino)pyrazolo[3,4-d]pyrimidine (Figure 4), was found to be a potent Mnk1 and Mnk2 inhibitor. It inhibits Mnk1 and Mnk2 with IC_50_ values of 0.7 and 0.8 μM respectively in an *in vitro* assay conducted with relatively low concentrations of ATP [65]. The compound also targets CK1 with similar potency as Mnk1 and shows potently inhibitory activity against other kinases including Aurora B, DYRK, SGK, BRSK2, and Lck within a low μM IC_50_ range [82]. Detailed cellular mechanistic studies revealed that CGP57380 substantially reduced eIF4G in the eIF4F complex and drastically inhibited eIF4E phosphorylation [83, 84]. It also decreased the expression levels of oncoprotein c-Myc and anti-apoptotic protein Mcl-1 [84]. Treatment of Jurkat T-cells with 40μM CGP57380 showed that eIF4E phosphorylation was completely blocked and TNFα production was inhibited by up to 75% [77], suggesting that Mnk may regulate TNFα mainly by modulating the translational efficiency of its mRNA. Interestingly, SHN-093, a methylated analogue of CGP57380 (Figure 4), was completely inactive against Mnk1/2 in both biochemical and cell-based assays, indicating the importance of 1-NH of pyrazolo moiety for Mnk inhibition. A binding model for CGP57380 to Mnk2 has been proposed (see below section). The model may offer a starting point for a medicinal chemistry optimisation program and the structure-activity relationship established would allow better understanding of the binding of inhibitors in the Mnk active site.

Isolated from *Cercosporidium henningsii*, cercosporamide was originally identified as a host-selective phytotoxin and broad spectrum antifungal agent [85]. Cercosporamide was later shown to inhibit a cell wall integrity pathway mediated through PKC1 (IC_50_ < 50 nM) [86, 87]. It was only recently discovered that cerosporamide is also a potent Mnk inhibitor, inhibiting Mnk1 and Mnk2 with an IC_50_ of 0.116 and 0.11 μM respectively [80]. However, it also inhibits a number of other kinases, including Jak3, GSK3β, ALK4 and Pim1, all in the low μM potency range [80]. Cercosporamide was the first Mnk inhibitor to show *in vivo* anti-tumour efficacy in human xenograft tumour models. Oral administration of a single dose of 20 mg/kg cercosporamide was able to significantly inhibit tumour growth in HCT116 colon carcinoma xenograft model. In a B16 melanoma mouse model cercosporamide also suppressed pulmonary metastases when dosed at 10 mg/kg (twice daily) or 20 mg/kg (daily) for 12 days, with minimal toxicity. Cercosporamide effectively blocked eIF4E phosphorylation at Ser209, suppressing cancer cell proliferation and colonization and leading to induction of apoptosis. As cerosporamide targets multiple kinases, it is important to dissect its exact biological mechanism of action.

## DESIGN OF SELECTIVE MNK INHIBITORS

**Figure 5:**
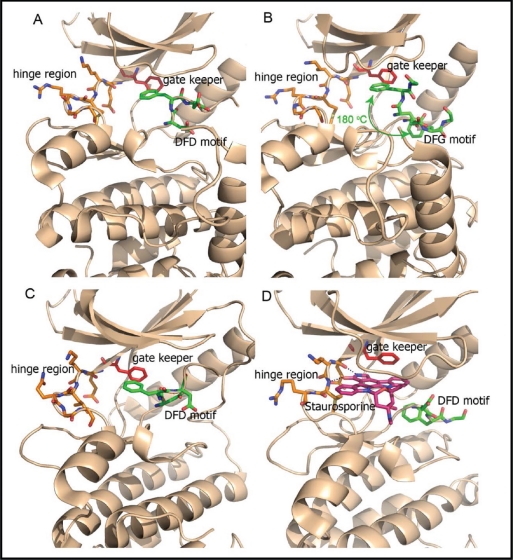
Ribbon plot (brown) displaying the crystal structures of Mnk kinase domain Residues around the DFD motif in either the DFG/D-IN or -OUT conformations are displayed as green sticks and the residues in the ATP-hinge region are shown as orange sticks. The gate keeper residues are indicated by red sticks. (A) The DFG/D-OUT conformation of wild-type Mnk2-KR is indicated with Phe227 and Asp228 poking into the ATP binding cleft (PDB 2AC3). The DFG/D-OUT conformation not only positions Phe227 and Asp228 in the ATP binding cleft, but also obstructs access to this cleft from the front. (B) In the Mnk2-KRD228G (Asp228Gly mutant), the DFG motif is found in both DFD/G-IN and DFD/G-OUT conformation and Phe227 flaps in/out shown by the green curly arrow (PDB 2AC5). (C) The DFG/D-OUT conformation of wild-type Mnk1-KR (PDB 2HW6), and (D) the structure of Mnk2-KRD228G–Staurosporine complex (PDB 2HW7). Staurosporine is displayed as a pink stick structure. The dashed lines indicate hydrogen bonds.

Mnks apparently have specific functions in cancer cells, which are redundant in the normal cells. These may be mediated through eIF4E's roles in mRNA translation and export, although it cannot be excluded that additional Mnk substrates are involved. It follows that in order to maximise the therapeutic margin of Mnk inhibitors, molecules with high selectivity for Mnk over other kinases are required. Structural studies reveal that the Mnk kinase domain is homologous to the family of Ca^2+^/calmodulin-modulated protein kinases (CaMK) [88]. However Mnk1/2 possess two distinct features: (1) their kinase domains contain a DFD motif (Asp191-Phe192-Asp193 in human Mnk1 and Asp226-Phe227-Asp228 in human Mnk2) which replaces the DFG (Asp-Phe-Gly) motif found in other protein kinases [61, 89, 90]; (2) the catalytic domain contains Mnk specific inserts (EVFTD in Mnk1 and EAFSE in Mnk2) not observed in other kinases. It has been suggested that the DFD motif makes it more difficult for ATP to access to the binding domain [61, 89]. Indeed, three-dimensional crystal structure analyses of the kinase regions of Mnk1 (Mnk1-KR) and Mnk2 (Mnk2-KR), as shown in Figure 5A and 5B, indicates that the DFD motif is rotated by 180° when compared to the DFG motif of other protein kinases. The Phe227 in the Mnk2-KR inserts into the ATP binding pocket, preventing ATP from entering this binding site (Figure 5A). This non-canonical arrangement of the DFD motif is referred to as the “DFG/D-OUT” conformation, as compared to the standard “DFG/D-IN” conformation found in other active kinases. Interestingly, the structure of Mnk2-KR (D228G), in which Asp228 was replaced with a glycine residue, showed that it could now adopt both DFG/D-IN and DFG/D-OUT conformations (Figure 5B). As shown in Figure 5C, the Mnk1-KR shows similar structural features to Mnk2-KR; however, the N-terminal lobe of Mnk2-KR is tilted by approximately 10 degrees, making the kinase binding pocket slightly more open to accommodate ATP or a small molecule inhibitor compared to Mnk1-KR. As the DFG/D-OUT conformation of Mnk2 is specific to the inhibitor-free protein kinase, Mnks are architecturally distinct from most other protein kinases, a feature which can be exploited for design of highly selective Mnk inhibitors. Analysis of the co-crystal structure of staurosporine in Mnk2-KR (D228G) [89] revealed that staurosporine binds in the canonical ATP active site in a fashion similar to its known binding mode in other protein kinases. The polycyclic ring system of staurosporine is sandwiched between the N-terminal and C-terminal lobes (Figure 5D). The 1-NH and 5-O atoms of staurosporine form hydrogen bonds to the backbone residues of Glu160 and Met162 in the hinge region (Figure 5D). The structural information is invaluable for the structure-based design of novel Mnk inhibitors.

The majority of small-molecule kinase inhibitors developed so far act as ATP competitors targeting the ATP binding site, with their respective kinases adopting an identical conformation to that used to bind ATP (the active conformation). These inhibitors are sometimes referred as type I kinase inhibitors [91]. The chemical scaffold of ATP-competitive inhibitors or type I inhibitors usually consists of planar heterocyclic systems that act as mimetics for the adenine moiety of ATP. They always contain characteristic adjacent hydrogen-bond-donor and -acceptor groups in the hinge region, the segment that connects the N- and C-terminal kinase domains, as well as hydrophobic functions. Many ATP competitive inhibitors have been successfully developed as therapeutics. However, due to the highly conserved structure of the ATP binding domain in most kinases, these inhibitors often suffer from cross-reactivity with other kinases, resulting in poor safety and sometimes severe side effects. Nevertheless a number of ATP competitive inhibitors have achieved good selectivity profiles by exploiting interactions with the non-conserved hydrophobic regions, where ATP binding is not involved, as well as interaction with the so-called ‘gatekeeper’ residue [92-95].

An alternative strategy for inhibitor design involves recognition of both the ATP binding cleft and the adjacent hydrophobic pocket created by the kinase activation loop. The activation loop is important in the regulation of kinase activity and in most protein kinases it is marked by conserved DFG and APE motifs at the start and end of the loop. Such inhibitors (sometimes termed type II inhibitors) are designed to make contact with residues of the hydrophobic pocket, which typically adopt the DFG-OUT conformation of an inactivated kinase. This unique hydrophobic pocket is also referred as an “allosteric site” [96]. As this binding site is less conserved among kinases than the ATP site, an inhibitor targeting this region can in principle achieve relatively high specificity. Indeed, such inhibitors, including imatinib and nilotinib, exhibit fewer side effects and good safety profiles in the clinic [91].

**Figure 6:**
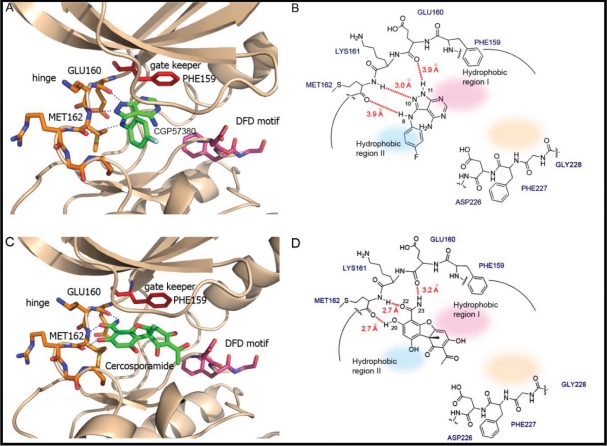
The binding models of Mnk inhibitors (A) Interactions of CGP57380 (green) within the ATP binding domain, and (B) schematic presentation of the corresponding interactions. (C) Interactions of cercosporamide (green) within the ATP binding domain, and (D) schematic presentation of the corresponding interactions. The DFD motif: pink sticks; Hinge region: orange sticks; Gate keeper: red sticks; Inhibitors: green sticks. Hydrogen bonds: dashed lines; Hydrophobic region I: shaded pink; Hydrophobic region II: shaded light blue; Allosteric pocket: shaded light beige. The likely binding posts were generated by Autodock 4.0 (http://w3.to/autodock). The structure of the Mnk2-KRD228G–Staurosporine complex (PDB2HW7) was used as the initial template. The dimensions of the active site box were chosen to be large enough to encompass the entire ATP binding pocket and allosteric site. Docking calculations were carried out using the Lamarckian genetic algorithm (LGA). A maximum number of 2,500,000 energy evaluations were used. Each docking experiment consisted of 100 independent runs.

The distinctive features of the DFD motif offer a unique opportunity for the discovery of highly selective Mnk inhibitors. To illustrate the structure-guided design approach involved, we performed *in silico* docking experiments for the Mnk inhibitors CGP57380 and cercosporamide. As the Phe227 residue in the DFD-OUT conformation projects into the ATP binding pocket to exclude the ATP or ligand from entering the binding site, experimental docking is a challenging task. For this reason, we used Mnk2 DFD-IN structure instead. Modelling studies of CGP57380 and cercosporamide, as shown in Figure 6, indicate that the overall binding modes of both inhibitors are very similar to that of staurosporine (Figure 5D). CGP57380 occupies the ATP-binding cleft between the two lobes of Mnk subunit (Figure 6A and 6B). The pyrazolo[3,4-*d*]pyrimidine moiety occupies the adenine subsite of the ATP-binding pocket, while the 4-fluoroaniline portion projects into the hydrophobic region II. The 1-NH, 2-N and 3-NH groups of pyrazolo[3,4-*d*]pyrimidine system form hydrogen-bonds with the backbone residues of Glu160, Lys161, and Met162 at the hinge region of Mnk2. Replacement of 1-NH with 1-NMe group would abolish the hydrogen-bond to Glu160, perhaps explaining why SHN-093 has significantly reduced Mnk inhibitory activity compared to CGP57380 [65]. The docking experiments also suggest that extension of the pyrazolo[3,4-*d*]pyrimidine heterocyclic scaffold, or introduction of an additional functional system at the 4-NH position, could generate hydrogen-bonds as well as hydrophobic interactions with the residues of the DFD motif. This should improve the potency and selectivity compared to CGP57380.

**Figure 7:**
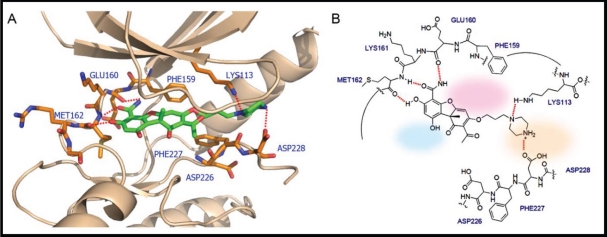
The interactions between Mnk2 and the proposed inhibitor which not only acts as ATP competitor but also interacts with the DFD motif (A) 3D view of the interactions. The inhibitor is shown in green sticks. Residues around the inhibitor are shown in orange sticks. Red dashed lines indicated the hydrogen bond interactions or electrostatic interactions. (B) Schematic presentation of the corresponding interactions. Hydrogen bonds: dashed lines; Hydrophobic region I: shaded pink; Hydrophobic region II: shaded light blue; Allosteric pocket: shaded light beige. The binding post was generated by the following procedures: the DFD/G-IN structure of the Mnk2 (PDB 2AC3) was used as the initial template; the conformation of Phe227 was modified to make the ATP binding pocket accessible for the inhibitor.

Cercosporamide exhibits a similar binding mode to CGP57380 (Figure 6C and 6D). It recognizes the ATP-binding domain through the characteristic hydrogen-bonding network, again involving the hinge region residues Glu160, Lys161, and Met162, whose backbone amide NH and carbonyl functions form hydrogen bonds with the 3-OH and 4-carboxamide of the phenyl portion of cercosporamide. The DFD motif residues would be an obvious candidate for full exploitation in order to achieve the optimal hydrogen-bonding and hydrophobic interactions. This can be achieved by some simple chemical modifications of the inhibitor compound. For example, introduction of butylpiperazine at the 7-OH position of cercosporamide, as shown in Figure 7, would appear to favour further contacts with the enzyme, involving hydrogen-bonding interactions with Asp228 and Lys113. Two further regions that are not involved in direct contacts with ATP, but which can be further exploited for inhibitor design, are a small hydrophobic pocket delineated by the gatekeeper residue Phe159 (hydrophobic I) at the base of the ATP-binding site and the hydrophobic region II which opens to the binding cleft. Manipulation and fine tuning of the structures by introducing the appropriate cyclic or acyclic functionalities would create an inhibitor that is capable of targeting both the ATP- and DFD-binding domains, thus achieving optimal potency and specificity.

## CONCLUSIONS

Significant advances have been made in validation of the Mnks as potential anti-cancer targets. This is an exciting prospect, given their roles in tumour cell biology and the fact they are dispensable for animal growth and development. The current state of knowledge about the structure of these enzymes strongly suggests that design of pharmacologic inhibitors that specifically inhibit Mnk kinase activity should be achievable. The task ahead is to discover inhibitors that not only possess high potency and specificity, but also favourable pharmaceutical properties. Such inhibitor compounds will serve as chemical biology tools for pharmacological target validation in terms of Mnk's role in regulation of Raf/MEK/ERK, PI3K/PTEN/Akt/mTOR and Jak/STAT pathways in cancers, as well as their functions required for normal physiological process. A deeper understanding of the biology and structure of Mnk would be invaluable in the ongoing discovery and development of new and better drugs for cancer treatment.
